# *APOE* – a genetic marker of comorbidity in subjects with morbid obesity

**DOI:** 10.1186/s12881-020-01082-2

**Published:** 2020-07-09

**Authors:** Per G. Farup, Helge Rootwelt, Knut Hestad

**Affiliations:** 1 Department of Research, Innlandet Hospital Trust, PB 104, N-2381 Brumunddal, Norway; 2 Department of Clinical and Molecular Medicine, Faculty of Medicine and Health Sciences, Norwegian University of Science and Technology, N-7491 Trondheim, Norway; 3 Department of Medical Biochemistry, Oslo University Hospital, N-0424 Oslo, Norway; 4 Department of Health- and Nursing Science, Faculty of Social and Health Sciences, Innland Norway University of Applied Sciences, N-2418 Elverum, Norway

**Keywords:** *APOE*, Comorbidity, CRP, Inflammation, Obesity, Psychosomatic disorders

## Abstract

**Background:**

In population-based studies, the genetic variability of the *APOE E* alleles have been associated with health outcomes. Health problems are common in subjects with obesity. This study explored associations between the *APOE E* alleles and comorbidity in subjects with morbid obesity.

**Methods:**

The study included consecutive subjects referred for evaluation of bariatric surgery with morbid obesity (defined as BMI > 40 or > 35 kg/m^2^ with complications related to obesity). The subjects followed a conservative weight loss program for 6 months before surgery and had a follow-up visit 12 months after surgery. Demographic data and a set psychosomatic scores (musculoskeletal pain, WHO-5 Well-Being Index, Rosenberg Self-Esteem Scale, Hopkins Symptom Check-list 10; Epworth Sleepiness Scale, and Fatigue Severity Scale) were collected, and blood samples were analysed for haematological and biochemical parameters and *APOE* alleles.

**Results:**

One hundred and forty subjects (men/women: 32 (23%)/108 (77%) with mean age 43.0 (SD 8.7) years and BMI 42.1 (SD 3.8) kg/m^2^ were included. One hundred and eight and 92 subjects had data after conservative treatment and 12 months after surgery, respectively. The prevalence of the *APOE* alleles were: *E2E2*: 1 (0.7%), *E2E3*: 13 (9.3%), *E2E4*: 4 (2.9%), *E3E3*: 71 (50.7%), *E3E4*: 47 (33.6%), and *E4E4*: 4 (2.9%). The prevalence rates were as anticipated in a Norwegian population. The weight loss during conservative treatment and after bariatric surgery was independent of *E* allele variability. *E2* was associated with a significant or clear trend toward improvement of all psychosomatic disorders. There was a significant fall in CRP during the two treatment periods with weight loss. *E2* and *E4* were significantly associated with high and low CRP, respectively, but no associations were seen between CRP and comorbidity.

**Conclusions:**

The most marked finding was the association between *E2* and improvement of all psychosomatic disorders. The positive and negative associations between CRP and *E2* and *E4,* respectively, could indicate effects on inflammation and immunological reactions.

## Background

The human *APOE* gene has three common allelic variants *E2*, *E3,* and *E4* encoded on chromosome 19. Depending on the genetic variability, the gene has been ascribed significant positive and negative health effects such as longevity and shortened lifespan, neurological and psychosomatic disorders, cognitive decline and Alzheimer disease, altered lipoprotein profile, atherosclerosis and cardiovascular disease, type II diabetes, changes in the immune response, oxidative stress, quality of life, physical activity, and obesity [1–4]. At large, *E4* has been ascribed unfavourable health outcomes and *E2* health-promoting effects [1, 5–11].

Obesity is a global health challenge due to the high prevalence of serious comorbidity [12–14]. The comorbidity of obesity (metabolic syndrome and type II diabetes, cardiovascular disease, musculoskeletal disorders and cancer) coincide in part with disorders associated with variability of the *APOE* isoforms. The associations between biomarkers and disease in subjects with obesity are, however, not always as expected, as exemplified by the protective effect of obesity on the outcome of cardiovascular disease (the obesity paradox) [15]. Except for a possible association between *E4* and obesity, little is known about associations between *APOE* variability and morbidity in subjects with obesity [2, 3].

The paramount aim of this study was to explore associations between the *APOE E* alleles and psychosomatic disorders and inflammatory markers in subjects with morbid obesity. The study aimed primarily at exploring the prevalence of *APOE E4* in subjects with morbid obesity and the effect of *E4* on conservative and surgically induced weight reduction. Secondary aims were to study the effects of all *APOE* alleles on weight and weight reduction, comorbidity and biochemical biomarkers. The associations between comorbidity and inflammatory markers and the *APOE* alleles were performed as post hoc analyses.

## Methods

### Study design

The data is a subset of the prospective cohort study in subjects with morbid obesity, the MO-BiPS study, (Morbid Obesity – Bio-Psycho-Social disorders) referred for evaluation of bariatric surgery at Innlandet Hospital Trust, Gjøvik, Norway [16–18]. After inclusion and before surgery, the participants followed a conservative weight-loss program for 6 months with dietary advice and increased physical activity (the first treatment period). A follow-up visit was scheduled 12 months after surgery (the second treatment period).

### Subjects

Consecutive subjects aged 18–65 years with morbid obesity (defined as BMI ≥ 40 kg/m^2^ or ≥ 35 kg/m^2^ with complications related to obesity) were from December 2012 to September 2014 included in the study. The doctors were responsible for the medical history and the physical examination, and decided necessary supplementary examinations. Blood samples were collected. The patints filled in paper-based questionnaires. Subjects with a somatic disease or psychiatric disorders in need of treatment and judged as unrelated to obesity, were excluded. A study nurse was responsible for the care of the patients and most of the practical work.

### Variables

The following variables were collected at inclusion:

- Gender, age (years), body mass index (BMI, kg/m^2^), smoking habits (never / previously / daily smokers), all regular use of drugs and present or previous somatic disorders including hypertension and diabetes (yes / no).
- Blood samples were analysed for standard haematological and biochemical variables, inflammatory markers [C-reactive protein (CRP), Interleukin-6 (Il-6) and Tumor Necrosis Factor (TNF)] and *APOE* genotype.
- Two questions assessed physical activity: Easy activity (not sweaty/breathless): none; < 1 h; 1–2 h; ≥3 h/week (score 0–3). Strenuous activity (sweaty/breathless): none; < 1 h; 1–2 h; ≥3 h/week (score 0, 3, 4, 5). Sum score physical activity 0–8.
- Musculoskeletal pain from six parts of the body (score 0–12).
- WHO-5 Well-Being Index (scores 0–100; scores ≤50 indicate low mood and scores ≤28 indicate likely depression) [19]
- Hopkins Symptom Checklist 10 measuring psychological distress (score 1–4; scores ≥1.85 indicate mental distress) [20].
- Fatigue Severity Scale. A Norwegian translation of the Fatigue Severity Scale was used (score 9–63; scores ≥36 indicate further evaluation) [21].
- Rosenberg Self-Esteem Scale. A validated Norwegian translation of the international questionnaire was used (score 10–40; values < 25 indicate low self-esteem) [22, 23].
- Epworth Sleepiness Scale. A validated Norwegian translation was used (score 0–24; normal 0–10; Mild 11–14; Moderate 15–18; Severe 19–25) [24].
- Sense of humour. A Norwegian version of the short form SHQ-6: “Attitudes toward humour” was used (score 6–24) [25].

Body weight and blood tests were available at inclusion, and before and 12 months after surgery for subjects following the scheduled plan.

### Statistics

The results have been reported as mean (with SD or 95% CI), and number with proportion (in percentage). Between groups compaisons were analysed with chi-square tests, t-test, and Pearson’s correlation analyses depending on the type of data. Independent predictors of demographics, comorbidity and biochemical biomarkers and *APOE* status were assessed with linear or logistic regression analyses and reported as B-value or Odds Ratio (OR) with 95% confidence interval (CI) and *p*-values. IBM SPSS Statistics for Windows, Version 25.0. Armonk, NY: IBM Corp was used for the analyses. *P*-values < 0.05 were judged as statistically significant. The genetic analyses were performed in previously collected blood samples [16–18]. The analyses were explorative, and no formal power calculation was performed.

## Results

Three hundred and fifty Caucasian subjects with morbid obesity were referred to the obesity unit during the inclusion period. One hundred and eleven were not included because study nurse was unavailable, 80 refused participation, 17 were excluded after erroneous inclusion, and two had had to be excluded because they did not fill in the questionnaires. The study included 140 subjects: men/women 32 (23%)/108 (77%) with mean age 43.0 (SD 8.7) years and BMI 42.1 (SD 3.8) kg/m^2^. One hundred and eight and 92 subjects had data on body weight and inflammatory markers immediately before bariatric surgery and 12 months after surgery, respectively.

The prevalence rates of the *APOE* alleles were: *E2E2*: 1 (0.7%), *E2E3*: 13 (9.3%), *E2E4*: 4 (2.9%), *E3E3*: 71 (50.7%), *E3E4*: 47 (33.6%), and *E4E4*: 4 (2.9%) giving prevalence rates of the *E2, E3,* and *E4* alleles of 6.8, 72.1 and 21.1% respectively. The *E3* allele was not significantly associated with any of the variables in the study (data not shown).

### Demographics and *APOE E2* and *E4* alleles

There was a non-significant trend towards an increased weight reduction in subjects with the *E4* allele after adjusting for age and gender. No other differences in the demographic variables were related to the *E2* and *E4* alleles. Table 1 gives the details.

**Table 1:** Associations between the subjects’ demographics and *APOE E4* and *E2* variants

Subject demographics (no is given if less than 140)	*APOE-E4* Present (no 55)	*APOE-E4* Absent (no 85)	Statistics *p*-value	Adjusted* p-value	*APOE-E2* Present (no 18)	*APOE-E2* Absent (no 122)	Statistics *p*-value	Adjusted* p-value
Gender (female)	43/55 (78%)	65/85 (76%)	*p* = 0.84		13/18 (72%)	95/122 (78%)	0.76	
Age (years)	42.6 (9.5)	43.3 (8.1)	*P* = 0.68		41.8 (8.4)	43.2 (8.7)	0.53	
Body weight (kg)	124.5 (19.3)	123.3 (18.4)	*P* = 0.72		126.2 (20.7)	123.4 (18.4)	0.56	
Height (cm)	171.6 (9.6)	170.7 (8.5)	*P* = 0.59		172.2 (9.2)	170.9 (8.9)	0.58	
BMI (kg / m2)	42.1 (4.0)	42.1 (3.7)	*P* = 0.99	−0.07 (−1.26;1.13) *p* = 0.91 **	42.4 (4.9)	42.1 (3.6)	0.73	0.00 (− 1.74; 1.74) *p* = 1.00 **
BMI reduction during the first treatment period (no 95)	3.2 (1.6)	2.9 (1.5)	*P* = 0.28	0.34 (−0.21; 0.98) *p* = 0.30	2.7 (1.4)	3.1 (1.6)	0.48	−0.28 (−1.22;0.66) *P* = 0.56
BMI reduction during the last treatment period (no 92)	11.0 (3.7)	10.5 (3.2)	*P* = 0.46	0.94 (−0.37;2.25) *p* = 0.16	10.0 (4.5)	10.8 (3.2)	0.44	−0.41 (−2.30;1.48) *p* = 0.67
BMI reduction total (no 92)	14.3 (3.8)	13.4 (3.5)	*P* = 0.21	1.36 (−0.04;2.75) *p* = 0.057	12.7 (5.1)	13.9 (3.3)	0.45	−0.72 (−2.76;1.32) *p* = 0.49

The results are given as mean (SD) or number (proportion). Chi-square and T-test were used for the comparisons. *Adjusted associations between the subject demographics (dependent variable) and *APOE E4* and *E2* variants were analysed with linear regression analyses adjusted for age, gender and BMI and reported as unstandardized coefficients (B-value) with 95% confidence intervals and *p*-values. ** Adjusted for age and gender

### Comorbidity and *APOE E2* and *E4* alleles

**Fig. 1:**
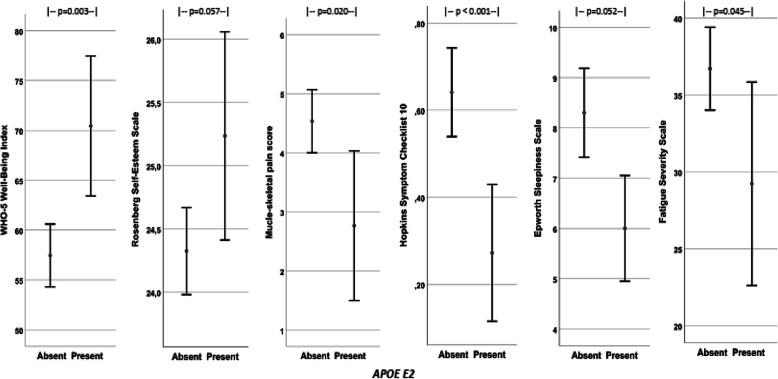
Shows the scores for the psychosomatic disorders in subjects with and without the *APOE E2* allele. The results are given as mean with 95% CI of the mean

Presence of the *E2* allele was favourably associated with all the psychosomatic variables (i.e. increased well-being and self-esteem, and reduced musculoskeletal pain, psychological distress, fatigue and sleepiness). Table 2 gives the details, and Fig. 1 shows the scores for psychosomatic comorbidity in subjects with and without *E2* with comparisons between the groups. Presence of *E2* was furthermore associated with hypertension in the adjusted analyses. No differences in comorbidity were seen between subjects with and without *E4*.

**Table 2:** Associations between comorbidity and *APOE E4* and *E2* variants

Subjects’ psychosomatic disorders (no is given if less than 140)	*APOE-E4* Present (no 55)	*APOE-E4* Absent (no 85)	Statistics *p*-value	Adjusted* p-value	*APOE-E2* Present (no 18)	*APOE-E2* Absent (no 122)	Statistics *p*-value	Adjusted* p-value
Diabetes (no 135)	12/54 (22%)	14/81 (17%)	*p* = 0.51	OR† 0.30 (0.55;3.33) *p* = 0.51	6/18 (33%)	20/117 (17%)	0.12	OR† 0.92 (0.77;8.1) *p* = 0.12
Hypertension (no 135)	19/54 (35%)	27/81 (33%)	*p* = 0.85	OR† 0.11 (0.051;2.48) *p* = 0.78	10/18 (56%)	36/117 (31%)	0.06	OR† 1.34 (1.21;12.1) ***p*** **= 0.022**
Physical activity (no 134)	4.5 (2.3)	4.5 (2.2)	*p* = 0.84	- 0.076 (−0.868;0.715) *p* = 0.85	5.1 (1.6)	4.4 (2.3)	0.15	0.666 (−0.465;1.798) *p* = 0.25
Musculoskeletal pain (no 137)	4.5 (3.0)	4.2 (2.9)	*p* = 0.66	−0.21 (− 0.81;1.23) *p* = 0.68	2.8 (2.5)	4.5 (3.0)	**0.020**	−1.74 (−3.23;-0.26) ***p*** **= 0.022**
WHO-5 Well-Being Index (no139)	58.2 (18.5)	59.8 (17.1)	*p* = 0.61	−1.60 (−7.73;4.52) *p* = 0.61	70.4 (14.1)	57.5 (17.5)	**0.003**	12.89 (4.22;21.56) ***p*** **= 0.004**
Hopkins Symptom Checklist 10 (no 135)	0.62 (0.57)	0.57 (0.53)	*p* = 0.58	0.06 (−0.13;0.25) *p* = 0.54	0.27 (0.32)	0.64 (0.56)	**< 0.001**	−0.36 (− 0.62;-0.09) ***p*** **= 0.009**
Rosenberg Self-Esteem (no 131)	24.7 (1.9)	24.3 (1.8)	*p* = 0.24	0.37 (−0.29;1.02) *p* = 0.27	25.2 (1.6)	24.3 (1.9)	0.057	0..87 (−0.08;1.81) *p* = 0.07
Fatigue Severity Scale (no 137)	36.6 (15.2)	35.2 (14.6)	*p* = 0.61	1.34 (−3.75;6.43) *p* = 0.52	29.2 (13.3)	36.7 (14.8)	**0.045**	−6.95 (− 14.22;0.32) *p* = 0.06
Epworth Sleepiness Scale (no138)	8.9 (5.1)	7.4 (4.3)	*p* = 0.06	1.52 (−0.09;3.12) *p* = 0.06	6.0 (2.1)	8.3 (4.9)	0.052	−2.22 (− 4.54;0.11) *p* = 0.06
Sense of humour (no 138)	19.6 (2.5)	18.9 (2.8)	*p* = 0.12	−0.71 (− 0.19;1.60) *p* = 0.12	18.9 (2.5)	19.2 (2.7)	0.70	−0.36 (−1.67;0.96) *p* = 0.60

The results are given as mean (SD) or number (proportion). Chi-square and t-test were used for the comparisons. *Adjusted associations between the subjects’ psychosomatic disorders (dependent variable) and *APOE E4* and *E2* were analysed with linear and logistic regression analyses adjusted for age, gender and BMI. The results are reported as unstandardized coefficients (B-value) or †Odds Ratio (OR) with 95% confidence intervals and *p*-values

### Biochemical biomarkers and *APOE E2* and *E4* alleles

**Fig. 2:**
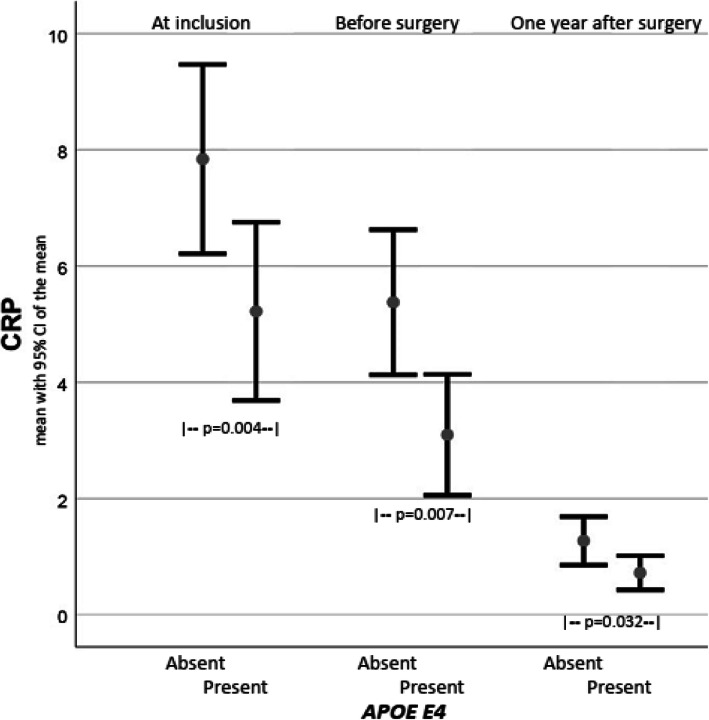
Shows the CRP values in subjects with and without the *APOE E4* allele at inclusion, before bariatric surgery (i.e. after 6 months with conservative weight loss) and 12 months after bariatric surgery

CRP was elevated at inclusion, was reduced after conservative induced weight loss, and continued to normalise after bariatric surgery. Presence of the *E4* allele was associated with lower CRP at all points of time. The presence of *E2* was associated with high CRP and HbA1c. Table 3 gives the details, and Fig. 2 shows CRP values in subjects with and without *E4* at inclusion, after conservative treatment and 12 months after surgery.

**Table 3:** Associations between biochemical biomarkers and *APOE E4* and *E2* variants

Biochemical biomarkers	*APOE-E4* Present (no 55)	*APOE-E4* Absent (no 85)	Statistics *p*-value	Adjusted* B-value (CI) *p*-value	*APOE-E2* Present (no 18)	*APOE-E2* Absent (no 122)	Statistics *p*-value	Adjusted* B-value (CI) *p*-value
Hb (no 139)	14.6 (1.1)	14.4 (1.1)	*P* = 0.23	0.24 (−0.06;0.54) *P* = 0.12	14.6 (1.2)	14.5 (1.1)	*p* = 0.57	0.09 (−0.36;0.53) *p* = 0.70
CRP at inclusion (no 139)	5.3 (4.6)	8.2 (7.1)	***p*** **= 0.004**	−2.98 (−5.06;-0.91) ***p*** **= 0.005**	9.6 (7.7)	6.7 (6.1)	p = 0.07	2.80 (−0.27;5.87) p = 0.07
CRP before surgery (no 108)	3.1 (2.8)	5.2 (4.4)	***p*** **= 0.007**	−1.65 (−3.12;-0.19) ***p*** **= 0.028**	7.1 (7.1)	4.0 (3.0)	*p* = 0.112	3.46 (1.48;5.42) ***p*** **= 0.001**
CRP 1 year after surgery (no 88)	0.7 (0.8)	1.3 (1.6)	***p*** **= 0.032**	−0.53 (−1.13;0.07) *p* = 0.08	2.3 (2.4)	0.9 (1.0)	*p* = 0.062	1.55 (0.77;2.33) ***p*** **< 0.001**
HbA1C (no 139)	5.9 (1.4)	5.8 (1.3)	*p* = 0.62	0.14 (−0.32;0.60) *p* = 0.54	6.5 (2.0)	5.8 (1.2)	*P* = 0.133	0.79 (0.14;1.44) ***p*** **= 0.018**
HDL cholesterol (no 139)	1.2 (0.4)	1.2 (0.3)	*p* = 0.69	0.03 (−0.08;0.13) *p* = 0.64	1.1 (0.3)	1.2 (0.3)	*p* = 0.25	−0.07 (− 0.22;0.09) *p* = 0.38
LDL cholesterol (no 139)	3.5 (0.8)	3.3 (0.8)	*p* = 0.08	0.25 (−0.04;0.55) *p* = 0.09	3.0 (1.0)	3.4 (0.8)	*p* = 0.06	−0.41 (− 0.83;0.02) p = 0.06
Il-6 (no 134)	2.5 (1.5)	3.0 (3.5)	*p* = 0.32	−0.52 (−1.55;0.51) *p* = 0.32	2.8 (1.4)	2.8 (3.1)	*p* = 0.95	−0.02 (−1.50;1.49) *p* = 0.98
TNF (no 135)	11.4 (28.8)	12.9 (49.5)	*p* = 0.84	−1.25 (− 16.3;13.8) *p* = 0.87	6.6 (2.5)	13.2 (45.7)	*p* = 0.54	−5.7 (−27.2;15.9) p = 0.60

The results are given as mean (SD). T-test was used for the comparisons. * Adjusted associations between the biochemical biomarkers (dependent variable) and *APOE E4* and *E2* were analysed with linear regression analyses adjusted for age, gender and BMI. The results are reported as unstandardized coefficients (B-value) with 95% confidence intervals and *p*-values

### Comorbidity and inflammation

*E2* and *E4* were associated with high and low CRP values, respectively. No associations were seen between *APOE* genotype and Il-6 and TNF. Table 4 gives the associations between the comorbidities and CRP, *E2* and *E4* adjusted for age, gender and BMI. The psychosomatic comorbidities and hypertension were associated with *E2* but not with CRP and *E4*. Diabetes was associated with CRP.

**Table 4:** Associations between comorbidity and CRP

Comorbidity	CRP*	CRP Adjusted†	*E2* Adjusted†	*E4* Adjusted†
Diabetes (no 135)	*r* = 0.096 *p* = 0.27	OR = 1.08 (1.004;1.16) ***p*** **= 0.039**	OR = 2.42 (0.71;8.21) *p* = 0.157	OR = 0.20 (0.72;5.08) *p* = 0.20
Hypertension (no 135)	*r* = − 0.096 *p* = 0.27	OR = 0.98 (0.92;1.06) p = 0.66	OR = 4.50 (1.34;15.11) ***p*** **= 0.015**	OR = 1.15 (0.49;2.73) *p* = 0.76
Musculoskeletal pain score (no 137)	*r* = −0.134 *P* = 0.12	B = -0.07 (−0.15;0.02) p = 0.12	B = -1.55 (−3.05;-0.05) ***p*** **= 0.043**	B = -0.025 (−1.29;0.78) *p* = 0.63
WHO-5 Well-Being Index (no139)	*r* = −0.034 *P* = 0.69	B = -0.21 (−0.70;0.29) *p* = 0.41	B = 13.1 (4.3;22.0) ***p*** **= 0.004**	B = -0.53 (−6.7;5.7) *p* = 0.87
Hopkins Symptom Checklist 10 (no 135)	*r* = 0.049 *P* = 0.57	B = 0.01 (−0.01;0.02) *p* = 0.35	B = -0.37 (−0.64;-0.10) ***p*** **= 0.009**	B = 0.05 (− 0.15;0.24) *p* = 0.64
Rosenberg Self-Esteem (131)	*r* = 0.005 *P* = 0.96	B = -0.01 (−0.06;0.05) *p* = 0.77	B = 1.01 (0.05;1.98) ***p*** **= 0.040**	B = 0.44 (−0.24;1.12) *p* = 0.20
Fatigue Severity Scale (no 137)	*r* = 0.021 *p* = 0.81	B = 0.08 (−0.34;0.50) *p* = 0.71	B = -6.9 (−14.4;0.5) *p* = 0.069	B = 0.70 (−4.57;5.97) *p* = 0.79
Epworth Sleepiness Scale (no138)	r = 0.019 *P* = 0.83	B = 0.07 (−0.07;0.20) *p* = 0.33	B = -2.10 (− 4.45;0.25) *p* = 0.080	B = 1.52 (− 0.13;3.17) *p* = 0.071
Sense of humour (no 138)	*r* = −0.028 *p* = 0.75	B = -0.03 (− 0.11;0.05) *p* = 0.42	B = -0.31 (−1.47;1.20) p = 0.85	B = 0.55 (− 0.39;1.49) *p* = 0.25

*The results are given as Pearson’s correlation coefficient (r). † Adjusted associations between the comorbidity (dependent variable) and CRP, *E2* and *E4,* age, gender and BMI were analysed with logistic and linear regression analyses. The results are reported as Odds Ratio (OR) and unstandardized coefficients (B-value) with 95% confidence intervals and *p*-values

## Discussion

The prevalence rates of the *APOE* alleles were consistent with those reported in both clinical and population-based studies from Scandinavia [1, 26–28].. The prevalence of *E4* might seem high, but considering the increasing south-north gradient and comparisons with studies from the same region, the prevalence rates were as expected [28, 29]. The observed distribution of the different genotypes was in complete agreement with the predicted genotypes according to Hardy-Weinberg equilibrium. In a case-control study with 198 obese and normal-weight subjects, the prevalence of *E4* was high in subjects with obesity [3]. In a review of pooled data from seven studies with 27,863 Caucasians, *E4* was associated with reduced BMI in individuals above 60 years of age but not in younger [2]. This study gives no support for an abnormal distribution of the *E* alleles in subjects with morbid obesity but is too small for a valid conclusion. Neither was the presence of any of the *E* alleles significantly associated with weight reduction after conservative treatment or bariatric surgery.

The main finding related to the *E4* allele was the significant association with low CRP. Similar findings have been reported in previous reports; the relative reduction of CRP has been estimated to 34% compared to subjects with zero *E4* alleles [30–33]. There was a marked reduction of CRP during both treatments periods. The significant differences in CRP between subjects with and without the *E4* allele persisted at all points of time. The reduction in CRP after weight loss was expected because CRP is strongly related to total fat mass, particularly in women, and most subjects in this study were women [34]. In the general population, high CRP is an independent predictor of cancer-, cardiovascular-, and overall mortality and ischemic stroke [35, 36]. Therefore, it seems contradictory that *E4*, which is associated with low CRP, is a predictor of cardiovascular disease and mortality, and reduced longevity [1, 37–40]. The same contradiction applies to cognitive functions and Alzheimer disease. CRP has been associated with subsequent cognitive impairment and age-related cognitive decline, whereas the *E4* allele, which was associated with low CRP, increases the risk of cognitive reduction and Alzheimer disease [5, 6, 41, 42]. The interactions between CRP, the *APOE* genotype, cardiovascular diseases and cognitive functions and dementia are confusing and might depend on gender, age, and disease such as obesity [43–46]. The association between CRP and atherosclerosis might be diminished in subjects with obesity [44]. In this study, after adjusting for age, gender and BMI, the *E2* allele was associated with high CRP-values after the two treatment periods and with hypertension. This was unexpected since *E2* has shown favourable health outcomes such as reduced low-density lipoprotein and increased survival [7]. Consequently, using CRP to estimate overall disease risk or prognosis may be misleading in subjects with morbid obesity.

Obesity with comorbidity (hypertension, metabolic syndrome, diabetes) leads to cardiovascular disease but protects against the clinical outcome such as short and long term mortality rates in subjects with cardiovascular disease [15, 47, 48]. The phenomenon has been referred to as the “obesity paradox”. Compared with normal-weight subjects, subjects with obesity have an unexplained preservation of vascular function with higher flow-mediated dilatation and reduced intima-media thickness [49]. The significance of inflammation on the obesity paradox is inconclusive. In one study, body fat protected against mortality in a subgroup with high CRP [50]. The association between *APOE* genotype and coronary disease was described as a puzzling paradox in 2006; the paradox is still not solved [51]. There is emerging evidence for plasma APOE as a risk factor for dementia, and to a lesser extent for ischemic heart disease, but the associations seem to be independent of *APOE* genotype [52, 53]. A recently published study suggests that other genetic factors might contribute to the obesity paradox [54].

In this study, the *E2* allele was associated with significant or clear trends toward the improvement of all the psychosomatic variables: Musculoskeletal pain, WHO-5 Well-Being Index, Rosenberg Self-Esteem Scale, Hopkin Symptoms Check-list 10; Epworth Sleepiness Scale, and Fatigue. Protection of *E2* against depression and reduced vulnerability to psychiatric and somatic diseases has also been reported in other studies [8–10]. In all, the *E2* allele seems to have a range of health-promoting effects. In addition to the psychosomatic effects, *E2* has been associated with longevity, delayed onset of cognitive decline, reduced LDL-cholesterol and reduced overall risk of cardiovascular diseases [1].

High and low CRP were associated with *E2* and *E4,* respectively. Inflammation has been linked to psychosomatic disorders [55–57]. Therefore, post hoc analyses were performed to rule out if inflammation could confound the associations between the psychosomatic disorders and the *APOE* alleles. *E2* was the only independent predictor of the psychosomatic disorders after adjusting for *E4*, CRP, gender, age and BMI (Table 4). CRP was associated with diabetes. These results support the reduced importance of CRP as a disease modifier in subjects with obesity [44].

### Strengths and limitation

Inclusion of consecutive subjects with morbid obesity referred for evaluation of bariatric surgery improves the validity of the results. However, since this group is only a minority of all subjects with obesity, the transferability of the results to all subjects with obesity is uncertain, and the results are not transferrable to subjects with normal weight. The study was too small for valid comparisons of the prevalence of the *E* alleles in subjects with morbid obesity and the general population, and for evaluation of the effects of the *E* alleles on weight loss, but no striking differences were observed. The association between low CRP and *E4* observed in this study is in accordance with other studies in subjects with and without obesity [30–33]. The lack of associations between CRP and comorbidity is known from other studies in subjects with obesity, which contrasts to findings in the general population [44]. Favourable effects of *E2* have also been reported in subjects with other disorders [1, 8–10]. Four subjects with the combination *E2:E4* were included in two groups, in the group with *E4* and the group with *E2,* and might have weakened the analyses. In all, the consistent findings indicate high external validity of the study.

## Conclusions

The most important finding was the marked improvement of all psychosomatic disorders related to the presence of the *E2* allele in subjects with morbid obesity. The *E2* allele has been ascribed health promoting effects, but the effects seemed more pronounced than in studies in non-obese groups. High and low CRP were associated with the *E2* and *E4* alleles respectively, but not associated with obesity-related disorders. The uncertain associations between CRP and comorbidity observed in other studies in subjects with obesity seem to imply also to those with morbid obesity. No notable effect of the *E* alleles was observed on BMI or reduction of BMI after conservative treatment or bariatric surgery.

## Data Availability

The raw datasets generated and analysed during the current study are not publicly available in order to protect participant confidentiality. Case report forms (CRFs) on paper are safely stored. The data were transferred to SPSS for statistical analyses and the data files are stored by Innlandet Hospital Trust, Brumunddal, Norway, on a server dedicated to research. The security follows to the rules given by The Norwegian Data Protection Authority, P.O. Box 8177 Dep. NO-0034 Oslo, Norway. The data are available on request to the authors.

## Abbreviations

BMI: Body Mass Index
CI: Confidence interval
CRP: C-reactive protein
Il-6: Interleukin-6
OR: Odds Ratio
SD: Standard Deviation
TNF: Tumor Necrosis Factor
WHO: World Health Organization
